# Artificial Intelligence in Oncology: A 10-Year ClinicalTrials.gov-Based Analysis Across the Cancer Control Continuum

**DOI:** 10.3390/cancers17213537

**Published:** 2025-11-01

**Authors:** Himanshi Verma, Shilpi Mistry, Krishna Vamsi Jayam, Pratibha Shrestha, Lauren Adkins, Muxuan Liang, Aline Fares, Ali Zarrinpar, Dejana Braithwaite, Shama D. Karanth

**Affiliations:** 1 Department of Medical Education, University of Miami Leonard M. Miller School of Medicine, Miami, FL 33136, USA; hverma@miami.edu; 2 Department of Comprehensive Dentistry, UT Health San Antonio School of Dentistry, San Antonio, TX 78229, USA; mistrys@livemail.uthscsa.edu; 3 Department of Surgery, College of Medicine, University of Florida, Gainesville, FL 32610, USA; k.jayam@ufl.edu (K.V.J.); pratibha.shrestha@surgery.ufl.edu (P.S.); ali.zarrinpar@surgery.ufl.edu (A.Z.); dbraithwaite@surgery.ufl.edu (D.B.); 4 Department of Health Science Center Libraries, University of Florida, Gainesville, FL 32610, USA; lauren.adkins@ufl.edu; 5 MD Anderson Cancer Center, Houston, TX 77030, USA; mliang2@mdanderson.org; 6 Department of Medicine, Division of Hematology/Oncology, College of Medicine, University of Florida, Gainesville, FL 32610, USA; aline.fares@medicine.ufl.edu; 7 University of Florida Health Cancer Center, University of Florida, Gainesville, FL 32610, USA

**Keywords:** machine learning, deep learning, informatics, digital health, AI applications

## Abstract

**Simple Summary:**

Artificial intelligence (AI) is increasingly being integrated into healthcare, enhancing the accuracy and efficiency of diagnosis and treatment. In oncology, where personalized care approaches are essential, AI offers unique opportunities to support clinicians and patients. In this study, we conducted a cross-sectional analysis of completed U.S.-based oncology clinical trials that involved AI technologies registered on ClinicalTrials.gov over the past decade. We identified 50 completed trials, most of which were interventional (66%). Machine Learning was the most frequently applied AI application, and most trials focused on cancer detection, particularly for colorectal cancer and unspecified cancer types. By mapping these studies across the Cancer Control Continuum, this study illustrates current progress and diverse applications of AI in oncology. It underscores the need for more transparent evaluation to ensure the clinical validity and the impact of AI tools on improving patient care.

**Abstract:**

Background/Objectives: Artificial Intelligence (AI) is rapidly advancing in medicine, facilitating personalized care by leveraging complex clinical data, imaging, and patient monitoring. This study characterizes current practices in AI use within oncology clinical trials by analyzing completed U.S. trials within the Cancer Control Continuum (CCC), a framework that spans the stages of cancer etiology, prevention, detection, diagnosis, treatment, and survivorship. Methods: This cross-sectional study analyzed U.S.-based oncology trials registered on ClinicalTrials.gov between January 2015 and April 2025. Using AI-related MeSH terms, we identified trials addressing stages of the CCC. Results: Fifty completed oncology trials involving AI were identified; 66% were interventional and 34% observational. Machine Learning was the most common AI application, though specific algorithm details were often lacking. Other AI domains included Natural Language Processing, Computer Vision, and Integrated Systems. Most trials were single-center with limited participant enrollment. Few published results or reported outcomes, indicating notable reporting gaps. Conclusions: This analysis of ClinicalTrials.gov reveals a dynamic and innovative landscape of AI applications transforming oncology care, from cutting-edge Machine Learning models enhancing early cancer detection to intelligent chatbots supporting treatment adherence and personalized survivorship interventions. These trials highlight AI’s growing role in improving outcomes across the CCC in advancing personalized cancer care. Standardized reporting and enhanced data sharing will be important for facilitating the broader application of trial findings, accelerating the development and clinical integration of reliable AI tools to advance cancer care.

## 1. Introduction

Over the past decade, the use of Artificial Intelligence (AI) in medicine has surged, ushering in a new era of rapid innovation in healthcare [1,2]. These AI advancements are fueled by the growing availability of multi-dimensional clinical data and its ongoing digitization [3,4]. Sophisticated AI technologies, including Machine Learning (ML), Natural Language Processing (NLP), Deep Learning (DL), and Computer Vision Systems, can now integrate and synthesize these data to support individualized patient care [5,6,7,8]. Oncology is well-poised to benefit from AI due to the heterogeneous nature of tumor presentation and cancer outcomes, positioning AI to support targeted cancer therapeutics [3,9]. Thus far, AI has been utilized to optimize cancer research, streamline clinical workflows, and enhance understanding of tumor biology [10,11,12,13]. Radiology and pathology have already integrated AI for image classification, nodule detection, tumor characterization, and surveillance of cancerous lesions [10,12,14,15,16].

Despite the rapid development of diverse AI technologies, the thorough evaluation of their performance in real-world medical settings remains limited [17,18,19]. To bridge the gap between technical success and practical applications, particularly on patient outcomes and treatment decisions, AI has increasingly carved out a niche within clinical trials [20,21]. These trials provide a structured setting to validate AI technologies prior to widespread adoption, exploring applications ranging from improving diagnostic accuracy to revolutionizing the clinical trials process itself [2,20,22,23]. ClinicalTrials.gov, the largest trial registry [24], was used in the present study to investigate AI applications in oncology clinical trials.

The National Cancer Institute’s (NCI) cancer control continuum (CCC), which delineates the various stages of cancer from its etiology to survivorship [25], was utilized as a framework to categorize these AI applications. NCI subcategories were also determined for each trial to provide further application specificity. Investigating trials along the CCC is especially valuable at this stage, as the comparative effectiveness of narrow versus general AI applications in healthcare remains an area of active investigation. Given that AI integration in healthcare is still evolving, oncology may currently benefit most from narrow AI approaches that target CCC touchpoints [3,26]. Previous studies have investigated AI applications registered on ClinicalTrials.gov in the fields of critical care [27], ophthalmology [28], pediatrics [29], cancer diagnosis [30], and general healthcare [31]. Additionally, systematic [32] and narrative reviews [3] have explored cancer-specific AI applications [33], post-diagnostic AI use [34], and broader implementation challenges [35]. However, this study is the first comprehensive analysis of completed, US-based AI-related oncology clinical trials registered on ClinicalTrials.gov over the past decade, evaluated using the CCC framework [25]. By leveraging ClinicalTrials.gov, we characterize study design, cancer types, and intended AI applications, offering insights into how AI is being planned and evaluated in clinical research.

## 2. Materials and Methods

This cross-sectional study analyzed data from ClinicalTrials.gov [36], to identify AI-related oncology clinical trials. The search strategy included terms for the following concepts: (1) Medical Subject Headings (MeSH) terms related to cancer; (2) AI-related MeSH terms sourced from the National Library of Medicine [37]; (3) location: United States; (4) trial period from 1 January 2015 to 18 April 2025; (5) Completed studies to ensure consistent, complete data for meaningful comparison. Trials with a recruitment status of “recruiting”, “not yet recruiting”, “active, not recruiting”, “unknown status”, “enrolling by initiation”, “terminated,” or “withdrawn” were excluded. Studies were also excluded if they did not focus explicitly on both cancer and AI. The ClinicalTrials.gov search query syntax is presented below, with further details provided in the Supplementary Materials.

Condition/disease,
(Cancer* OR oncology OR leukemia* OR sarcoma* OR tumor* OR tumour* OR melanoma* OR neoplasm* OR neoplasia OR carcinoma* OR adenocarcinoma* OR lymphoma*),
AND,
Other Terms: (“Artificial Intelligence” OR “AI System” OR “Intelligent Systems” OR “Machine Learning” OR “Deep Learning” OR Autoencoder OR “Large Language Model” OR “Natural Language Processing” OR “Neural Network” OR “Convolutional Neural Network” OR “Generative AI” OR “Generative Pre-trained Transformer” OR “ChatGPT”),
AND,
(United States),
AND,
(Completed Studies),
AND,
(Study Start Date ≥ 1 January 2015).

**Figure 1:**
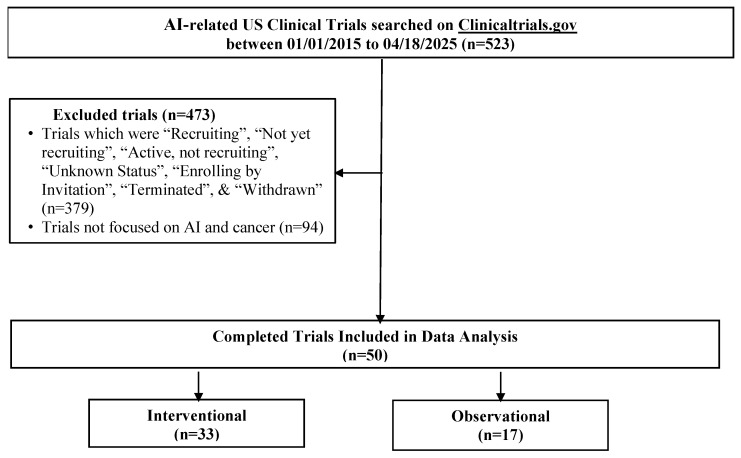
Flow diagram of AI-related oncology clinical trials identified from ClinicalTrials.gov.

Demographic and trial-level data, including age, sex, cancer type, AI term(s), study design, and trial characteristics, were manually abstracted from ClinicalTrials.gov by one author (HV) and independently verified by two authors (KV and SK) for accuracy. Fifty trials met the inclusion criteria (see Figure 1 and the full search query provided in the Supplementary Materials for the full list of terms).

Each study was classified as either an interventional or an observational study. For interventional trials, additional variables abstracted included allocation method (Randomized, non-Randomized, and not applicable [N/A]), interventional model assignment (single group, crossover, parallel, factorial), and masking (non-masked, single, double, triple). For observational trials, we recorded time perspective (prospective, retrospective) and observational model (cohort, case–control, family-based, and other). Finally, each trial’s AI application was categorized by the CCC framework domains: etiology, prevention, detection, diagnosis, treatment, and survivorship. Trials with multiple NCI subcategories were counted in each relevant domain. To reflect AI’s evolving role in oncology, we adapted the original CCC framework [25] by expanding definitions and incorporating terms relevant to contemporary study design and AI use. For example, we included imaging and fecal immunochemical testing (FIT) under detection in AI-enhanced screening and risk stratification. These modifications were based on iterative reviews of trial objectives, interventions, and clinical relevance. Additionally, the number of AI technologies in each trial type was characterized using a previously published AI taxonomy [38]. No institutional review board approval was necessary for this analysis of deidentified, publicly available data.

## 3. Results

**Figure 2:**
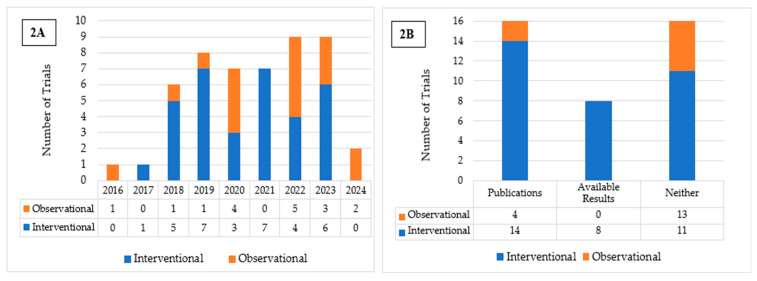
(**A**) Distribution of Interventional and Observational Trials by Year of Registration. (**B**) Publication and Results Reporting Status of Interventional and Observational Trials.

**Figure 3:**
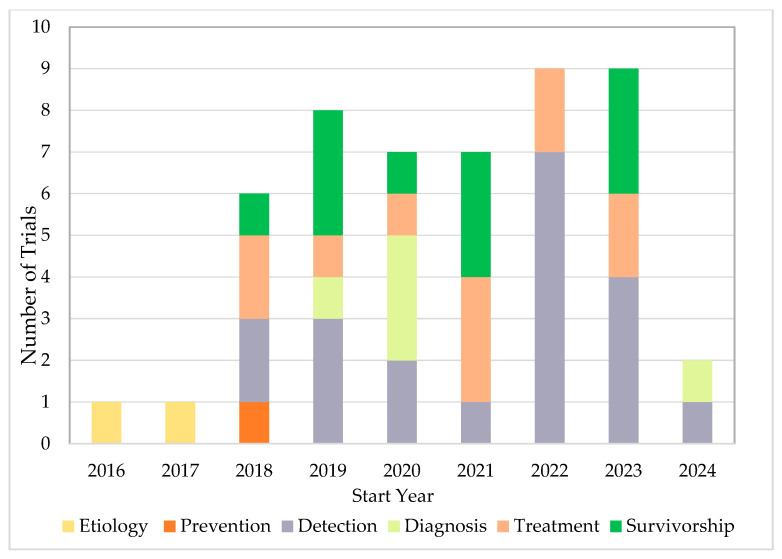
AI-Related Oncology Trials Initiations Across the CCC Stages.

Fifty completed U.S.-based clinical trials focusing on AI applications in oncology during the 10-year search period from 2015 to 2025 were identified (Figure 1). 33 (66.0%) were interventional and 17 (34.0%) were observational (Table 1). As illustrated in Figure 2A, the number of registered clinical trials increased from 2016, with interventional trials peaking in 2019 (n = 7), 2021 (n = 7), and 2023 (n = 6), and observational trials peaking in 2020 (n = 4) and 2022 (n = 5). Trial numbers declined in 2024, and no eligible trials were identified in 2025, likely reflecting the time required for trial completion and result reporting. Figure 3 illustrates the distribution of AI-related oncology trials across the CCC stages, with a peak in trial initiations between 2019 and 2023. Early in the decade, trials were etiology-focused, while post-2017 saw a marked surge in detection, treatment, and survivorship trials, reflecting a shift toward clinically applied AI research.

Among the interventional trials, eight (24.2%) had available results and 14 (42.4%) had associated publications listed on ClinicalTrials.gov (Table 1; Figure 2B). In contrast, none of the observational trials presented available results, and only four (23.5%) listed associated publications (Table 1; Figure 2B). Interventional trials predominantly enrolled fewer than 100 (33.3%) participants or between 100 and 500 (45.5%), with a median enrollment of 194. Observational trials frequently enrolled either fewer than 100 participants (35.3%) or more than 1000 (35.3%), resulting in a higher median enrollment of 355. Participants in both trial types were predominantly adults and older adults (97.0% and 70.6%, respectively), had similarly high rates of unrestricted gender recruitment (81.8% and 82.4%, respectively), and were mostly single-center (63.6% and 58.8%, respectively). For both trial types, a substantial proportion of studies were funded by sources other than the NIH and Industry, such as universities, individuals, and organizations, comprising 72.7% of interventional and 47.1% of observational trials. More interventional trials employed FDA-regulated devices (n = 15) than observational trials (n = 3). Eight interventional and all three observational trials involved unapproved/uncleared devices. Among interventional trials, the most reported cancer category included either multiple or unspecified cancer types (33.3%), followed by colorectal (30.3%) and breast cancer (12.1%). Similarly, in observational trials, multiple cancer types or unspecified (29.4%) were most common, followed by colorectal (17.6%), lung (11.8%), prostate (11.8%), and skin cancer (11.8%) (Table 1).

Among interventional trials, 22 (66.7%) trials had randomized allocation, one (3.0%) had non-randomized allocation, and ten (30.3%) did not fall into either category (Table 2). The majority employed a parallel assignment (n = 19, 57.6%); ten (30.3%) used single group assignment, while two each (6.1%) used crossover or factorial assignments. Most trials had no masking (n = 20, 60.6%), while the remainder included single (n = 6, 18.2%), double (n = 4, 12.1%), or triple (n = 3, 9.1%) masking.

Within the observational trials group, nine (52.9%) were cohort studies, three (17.6%) were case–control, one (5.9%) was family-based, and four (23.5%) fell into other types of studies. Regarding time perspective, the majority were prospective studies (n = 9, 52.9%), followed by retrospective studies (n = 5, 29.4%), cross-sectional (n = 1, 5.9%), and two classified as other (11.8%).

The most common AI subdomain across both trial types was ML (Table 3). Among the interventional trials, specific ML algorithms consisted of Convolutional Neural Networks (CNN; n = 1) and DL (n = 4), while seven trials did not specify the ML algorithm used. In observational trials, the specific ML algorithms included CNN (n = 1), Deep Ensemble (n = 1), and Deep Neural Network (n = 1), whereas 12 trials did not specify the ML algorithm used.

The next most frequently applied AI subdomain was NLP (Table 3). Three of the interventional trials listed specific NLP applications, including a Chatbot (n = 1) and Sentiment Analysis (n = 2). None of the observational trials specified the NLP application used. One interventional trial used Computer Vision with an application in Intelligent Real-Time Image Segmentation. Two interventional trials also reported the use of an integrated AI system with the application of an Intelligent System. Most trials did not specify the AI subdomain (17 interventional trials and eight observational trials).

Both interventional and observational trials targeted multiple stages of the CCC. The corresponding NCI subcategories and examples of specific study applications in each stage are summarized in Table 4.

**Figure 4:**
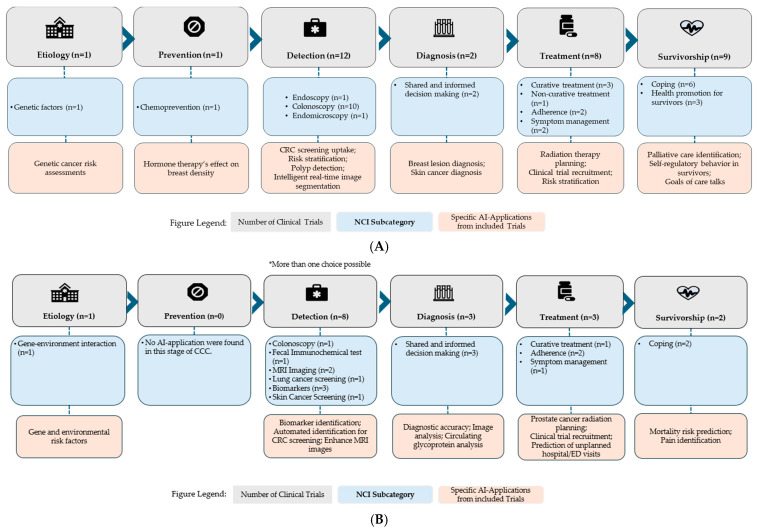
(**A**) Number of Interventional clinical trials across the Cancer Control Continuum stages. (**B**) Number of Observational clinical trials across the Cancer Control Continuum stages. * More than one choice possible indicates that some trials were classified under multiple CCC stages if their AI applications spanned more than one domain.

Across the CCC, interventional trials targeted various phases. (Figure 4A). One interventional trial focused on etiology, using AI to study genetic factors, and another targeted chemoprevention. Detection was the most targeted CCC component, with 12 trials applying AI in colonoscopy (n = 10), endoscopy (n = 1), and endomicroscopy (n = 1). Two trials focused on diagnosis, using AI to support shared and informed decision-making, while eight trials targeted treatment, including curative treatment (n = 3), adherence (n = 2), symptom management (n = 2), and non-curative treatment (n = 1). Nine trials targeted survivorship, including coping (n = 6) and health promotion (n = 3).

Observational trials similarly covered the CCC, with a more limited scope. One focused on etiology to investigate gene-environment interactions (Figure 4B). None targeted prevention. Detection was again the most frequently targeted, with eight trials focusing on AI applications in colonoscopy (n = 1), FIT (n = 1), MRI imaging (n = 2), lung cancer screening (n = 1), biomarkers (n = 3), and skin cancer screening (n = 1). Three trials focused on diagnosis, utilizing AI to support shared and informed decision-making. Three trials targeted curative treatment (n = 1), adherence (n = 2), and symptom management (n = 1). Finally, two trials focused on survivorship, both involving coping. AI Applications of all included trials are provided in Table 5 with additional information in Supplementary Table S1.

## 4. Discussion

This cross-sectional analysis of trials registered on ClinicalTrials.gov [36], over the past decade, highlights the applications of AI in interventional and observational oncology trials. To our knowledge, this is the first study to provide a comprehensive assessment of completed AI-related oncology clinical trials across the CCC.

### 4.1. Trial Characteristics

This analysis highlights the growing number of AI-related oncology trials, indicating increased interest in integrating AI across the CCC. These trials investigated a variety of interventions, including FDA-regulated devices, some of which were not yet approved or cleared. Study designs included parallel, single-group, crossover, and factorial designs. Other designs, such as family-based and case–control studies, as well as a small subset that did not fit traditional categories, illustrate the use of non-standard methodologies. Single-center studies were common, while multicenter trials were less frequent [40].

Despite the growing number of trials, few reported results or had associated publications, highlighting a need for improved transparency. Robust reporting is essential to achieve a comprehensive understanding of AI algorithms, including their parameter configurations, foundational theories, and procedural implementations. Moreover, to evaluate clinical efficacy, it is critical that results related to patient outcomes, such as diagnostic accuracy and treatment efficacy, are systematically reported on ClinicalTrials.gov. Standardized reporting frameworks, such as CONSORT-AI for RCTs and SPIRIT-AI for interventional protocols [41], along with PROBAST-AI [42] for bias assessment, and QUADAS-AI [43], offer guidance to improve methodological rigor, reduce bias, and support reproducibility. Greater adherence to these guidelines will be essential for advancing trustworthy AI integration in oncology.

### 4.2. AI Applications Across the CCC

Most trials targeted multiple or unspecified cancer types, reflecting the broad applicability of AI in oncology. Among site-specific cancers, colorectal cancer (CRC) was the most frequently studied, likely due to its prevalence and availability of conducive data for AI-driven approaches. To illustrate the range of AI applications, we highlight representative trials for each CCC stage below, demonstrating how AI has been applied across the cancer care continuum.

#### 4.2.1. Etiology

A limited number of trials focused on etiology highlights a potential gap in the application of AI across the CCC. Despite AI’s potential to help uncover genetic and environmental contributors to cancer, its application in etiology-related cancer trials remains limited. One interventional trial (NCT03511690) evaluated the use of an intelligent tutoring system, “BReast CAncer Genetics Intelligent Semantic Tutoring” (BRCA Gist), designed to promote participation in genetic cancer risk assessment among underserved Latina and Black women at hereditary risk of ovarian and breast cancer. The intervention uses AI-supported avatars to deliver personalized tutoring on breast cancer, metastasis, risk factors, genetic testing, and its implications. The avatars incorporate text, graphics, and video and are available in multiple ethnicities to provide culturally tailored education. The system employs Latent Semantic Analysis to interpret user responses and generate personalized feedback. A related publication reported that participants using BRCA Gist demonstrated improved understanding of breast cancer genetics and made more informed decisions about genetic testing compared to controls [44]. This trial illustrates how NLP and adaptive AI technologies can enhance shared decision making and support genetic risk counseling. The observational trial (NCT03174574) applied ML to survey-based data to identify risk factors for pancreatic and melanoma cancers in individuals with hereditary predispositions. The study demonstrated the utility of AI in analyzing gene-environment interactions to advance etiological understanding in high-risk populations. Both trials underscore the untapped potential of AI to inform cancer prevention and risk assessment through enhanced etiologic insights.

#### 4.2.2. Prevention

Like the limited number of trials focused on etiology, cancer prevention remains an underrepresented area for AI-driven interventions, with only one interventional trial identified in this domain. This randomized trial (NCT03063619) evaluated afimoxifene, a hormone therapy, to reduce breast cancer risk in women with dense breast tissue. A secondary objective compared two breast density measurement tools: Cumulus, a semi-automated software using reader-defined thresholds, and Volpara, which applies X-ray physics and ML to generate volumetric breast measurements for breast cancer prevention. The results reported that Volpara produced more consistent and less variable measurements, while Cumulus was more sensitive to larger changes in breast density [45]. This trial illustrates the potential of ML-based tools to enhance chemoprevention strategies in high-risk populations.

#### 4.2.3. Detection

Detection was the most frequently targeted touchpoint of the CCC, with 12 interventional and 8 observational trials focused on this domain. Most interventional trials applied AI to enhance CRC detection. For example, one trial (NCT06621225) evaluated the performance of a CNN-based computer-aided detection (CAD) system called “GI Genius”, which analyzes real-time endoscopic images to better detect polyps. Another trial (NCT03925337) assessed a DL-based research software developed using a database of polyp images and operation videos to detect polyps and adenomas during colonoscopy procedures. A related publication reported that AI-assisted colonoscopy significantly reduced the adenoma miss rate compared to standard procedures [46]. Another trial (NCT03867409) explored the use of virtual human technology to promote CRC screening by delivering culturally tailored messages to at-risk communities. The high number of AI-related colonoscopy trials highlights AI’s increasing influence on gastrointestinal endoscopy [47]. This field is well-suited for AI integration due to its reliance on real-time image and video analysis [48] as well as its potential to utilize virtual platforms that enhance patient engagement and screening uptake.

Among observational trials, two observational trials also focused on improving CRC detection. One validated Freenome, an ML-based blood test for CRC biomarkers (NCT04369053), while the NCT05383976 trial used ML to risk-stratify patients who had not undergone colonoscopy and provided navigator support to increase screening uptake. A third observational trial (NCT05126173) focused on skin cancer detection, clinically validating “DERM”, a Deep Ensemble algorithm for identifying malignancies from lesion images. Together, these trials underscore the diverse applications of AI in oncology, ranging from biomarker discovery and risk stratification to image-based detection, and demonstrate its potential across multiple cancer types and care pathways. Many trials focused on CRC, likely because colonoscopy is well-suited for AI integration, particularly in real-time image analysis, due to the availability of large annotated datasets, standardized screening protocols, and the clinical importance of early polyp detection, all of which make CRC an ideal candidate for AI integration.

#### 4.2.4. Diagnosis

Diagnosis was a less frequently targeted component of the CCC. An innovative approach involved a DL-based computer-assisted detection (NCT04551105) and diagnosis system for the interpretation of breast ultrasound images of cancer lesions, exploring how an AI-assisted device may enhance diagnostic accuracy and reduce reading time compared to non-AI image reading [49].

Recent efforts in observational research have explored tools like DermDx (NCT06463860), a DL-based reading aid designed to support primary care physicians in skin cancer diagnosis, which demonstrates AI’s potential to serve as the first point of contact in cancer detection [50]. Another trial (NCT05147389) sought to clinically validate a CNN-based model trained on cholangioscopy images to distinguish malignant from benign bile duct lesions. An associated publication reported that the model achieved statistically high accuracy in differentiating lesion types [51]. These trials demonstrate that current AI applications in diagnosis are leveraging image-based data to target various cancers. They also highlight AI’s potential to improve diagnostic accuracy, reduce clinical burden, and expand access to expert-level diagnostic support.

#### 4.2.5. Treatment

AI applications in cancer treatment focused on curative and non-curative care, treatment adherence, and symptom management. An example includes “CancerLife” (NCT03371147), a mobile application that supports cancer patients and caregivers by collecting patient-reported symptoms and analyzing emotional tone using IBM Watson’s NLP-based Sentiment Analysis. While “MatchMiner” (NCT06888089), uses an ML and NLP-powered platform to integrate genomic and clinical data to match patients to biomarker-driven therapeutic trials, aiming to optimize treatment decisions and clinical trial enrollment. “Penny” (NCT05113264), an NLP-assisted chatbot, reminds patients to promote oral chemotherapy adherence and symptom management. This intervention reflects an interactive approach to promote chemotherapy adherence through direct patient engagement [52]. “Ethos” (NCT05030454), an AI-powered device that adapts radiation treatment plans in response to anatomical changes between therapy sessions, represents a promising avenue to enhance the precision and safety of radiotherapy delivery.

Observational trials in cancer treatment are applying AI in various ways, including symptom monitoring, treatment adherence, personalized planning, and clinical trial matching (e.g., NCT04441775, NCT06561217). The “SHIELD-RT” trial (NCT05122247) evaluated a previously validated ML model to predict unplanned hospital admissions and emergency department visits among cancer patients receiving systemic therapy [53]. This model supports personalized care planning and timely clinical interventions by identifying high-risk patients. Such applications reflect a shift toward more precise and efficient patient-centered care.

#### 4.2.6. Survivorship

AI applications in survivorship trials addressed emotional coping and health promotion among cancer survivors. ML algorithms can identify oncology patients at high risk of six-month mortality (e.g., NCT03984773) [54]. Patients identified as high-risk triggered behavioral nudges, prompting oncologists to initiate serious illness conversations. An associated publication reported significantly more conversations in the intervention group than in the control group, illustrating how AI can influence clinician behavior and promote goal-concordant end-of-life care [55]. The NCT05069519 trial aimed to promote healthy behaviors among cancer survivors through personalized exercise regimens. The study incorporated Fitbits for activity tracking and a private Facebook group for health education. NLP and text mining were used to analyze posts, assess sentiment and engagement, and deliver weekly personalized feedback, demonstrating the potential of combining AI and social media to support behavioral change in survivorship care. NCT04458168 evaluated the feasibility and acceptability of “Purposeful”, a mobile application that supports behavior change and prompts self-reflection among survivors. The app uses ML to personalize content based on user interactions, displaying how AI can be tailored to individual needs in survivorship care.

Among observational trials, NCT04442425 explored coping among cancer patients by evaluating facial recognition technology for pain monitoring. Participants submitted audio-visual pain reports via a mobile application, and researchers used ML and NLP to analyze facial expressions and speech related to pain. This approach highlights a novel use of AI for real-time pain monitoring. These trials demonstrate the expanding use of AI in survivorship, particularly in promoting behavior change, to support advanced care planning, and monitoring pain to provide personalized long-term cancer care.

### 4.3. Barriers to AI Integration in Oncology

Despite the transformative potential of AI in cancer care, several barriers hinder its adoption in clinical practice. AI models require large, diverse, and high-quality datasets for effective training. However, obtaining such data can be challenging due to concerns regarding data confidentiality, ownership, and institutional data silos [56,57]. These data challenges are further compounded in surgical contexts where variation in operative techniques, the substantial time burden associated with annotating surgical imaging and video data, and hesitancy to record and distribute such data limit the creation of comprehensive datasets [58]. Furthermore, ML technologies rely on continuous data input to improve performance, which can be difficult to maintain in real-world clinical settings [57]. Data security and patient privacy are additional concerns as AI applications handle sensitive information, necessitating informed consent and strict data protection measures [56,57,58].

Algorithmic bias is a major limitation in AI applications. Bias can arise when training datasets fail to generalize to large, diverse patient populations [56]. For example, one study found that an AI algorithm diagnosed skin cancer more accurately in lighter skin tones than in darker ones, emphasizing the need for representative datasets in model development [59]. Another key barrier to clinical adoption is the lack of transparency in AI model decision making. Often described as “black boxes”, these models produce outputs that are difficult to interpret or validate, complicating accountability in cases of clinical error, which poses an ethical and legal challenge in patient care [57,60]. Regulatory challenges, such as FDA approval for adaptive AI algorithms [61], further hinder integration due to difficulties in validating evolving models. However, most included trials did not involve applications currently seeking approval, reflecting their early-stage development.

It is clear that careful attention to AI-related concerns is essential. This study highlights that publicly available trials [36] should provide detailed information on training datasets and the specific models or algorithms employed to enhance transparency and reproducibility. Moreover, rigorous testing through prospective randomized trials is essential for clinical validation; however, this study design was notably underrepresented among the included trials. Transparent reporting and robust study design remain critical for the safe and effective integration of AI into oncological practice.

### 4.4. Limitations

This study had several limitations. First, the MeSH terms used were extracted from the National Library of Medicine’s defined list for AI and cancer. Although we sought to ensure this list was comprehensive, more specific or emerging AI and cancer terms may not have been included, potentially leading to an underestimation of the AI-related trials registered on ClinicalTrials.gov. Second, the analysis was limited to ClinicalTrials.gov, which, although one of the most used clinical trial registries, does not represent the global scope of oncology trials. Moreover, we further restricted our analysis to studies conducted in the United States. Consequently, AI-related oncology trials conducted in other countries or registered in international trial databases were not included, which may limit the generalizability of our findings and underrepresent global efforts in this area. Future studies should incorporate multiple international trial registries to provide a more comprehensive overview of global AI applications in oncology. Additionally, inconsistent reporting of trial outcomes such as clinical efficacy, safety, and adherence restricted our ability to assess the implementation readiness of AI tools in oncology trials. Finally, this study focused solely on extracting data from trials marked as complete to obtain the most detailed information available.

## 5. Conclusions

This study is the first to provide a comprehensive overview of US-based AI-related oncology clinical trials registered as complete on ClinicalTrials.gov between 2015 and 2025. Although ClinicalTrials.gov is publicly accessible, relatively few trials post results or have peer-reviewed publications, which limits understanding of how these tools perform in practice. As a result, a gap remains between AI-based predictive modeling tools and the real-world practice of clinical oncology care. Addressing this gap will require more rigorous, multicenter trial designs, broader dissemination of findings, and standardized, transparent reporting of AI methodology in accordance with existing reporting frameworks, such as the CONSORT-AI and SPIRIT-AI guidelines. The current trial landscape reflects a rapidly evolving and innovative field with significant potential to transform cancer care across the continuum. However, to enhance the generalizability and global relevance of these findings, the focus should shift to diverse populations and global settings.

## Figures and Tables

**Table 1:** Characteristics of completed AI-related oncology clinical trials from Clinicaltrials.gov.

Study Characteristics	All n = 50	Interventional n = 33	Observational n = 17
Results			
Results Available No Results Available	8 42	8 25	0 17
Published Studies Available No Published Studies Available	18 32	14 19	4 13
Participant Enrollment			
≤100 100–500 500–1000 >1000	17 18 3 12	11 15 1 6	6 3 2 6
Median	198	194	355
Age group			
Adults and older adults Children, adults, and older adults Older adults (>65)	44 5 1	32 0 1	12 5 0
Gender			
Female only Male only Both	7 2 41	6 0 27	1 2 14
Center			
Single center Multi-center	31 19	21 12	10 7
Funder Type			
NIH Industry Other *	3 15 32	1 8 24	2 7 8
FDA Regulation Status			
Regulated Device Regulated + Unapproved/Uncleared	18 11	15 8	3 3
Cancer type			
Bile Duct	1	0	1
Blood	1	1	0
Breast	4	4	0
Colorectal	13	10	3
Esophagus	2	1	1
Gastrointestinal	2	2	0
Gynecologic	3	2	1
Head and Neck	1	1	0
Lung	2	0	2
Prostate	2	0	2
Skin	3	1	2
Multiple or Unspecified	16	11	5

* Other funder types include individuals, universities, and organizations.

**Table 2:** Characteristics of interventional and observational AI-related oncology trials from ClinicalTrials.gov (2015–2025).

Characteristics	Number of Trials
Interventional (n = 33)	
Allocation	
Randomized	22
Non-randomized	1
N/A *	10
Intervention model	
Single group assignment	10
Crossover assignment	2
Parallel assignment	19
Factorial assignment	2
Masking	
Non-masked	20
Masked	13
Single	6
Double	4
Triple	3
Observational (n = 17)	
Observational model	
Cohort	9
Case–Control	3
Family-Based	1
Other	4
Time perspective	
Cross sectional	1
Prospective	9
Retrospective	5
Other	2

* N/A denotes Not applicable to either category.

**Table 3:** Applications of artificial intelligence in oncology clinical trials.

AI Domain (n) *	Subdomain (n) *		Keyword (n)
Interventional Trials			
Learning (n = 12)	Machine Learning (n = 12) NCT03984773, NCT06888089, NCT06621225, NCT04551105, NCT04867850, NCT04535414, NCT03746392, NCT04277650, NCT05611151, NCT04458168, NCT04195646, NCT03063619		Convolutional Neural Network (n = 1) NCT06621225
			Deep Learning (n = 4) NCT06621225, NCT03925337, NCT04551105, NCT04195646
Communication (n = 5)	Natural Language Processing (n = 5) NCT06888089, NCT05113264, NCT03371147, NCT05069519, NCT03746392		Chatbot (n = 1) NCT05113264
			Sentiment Analysis (n = 2) NCT03371147, NCT05069519
Perception (n = 1)	Computer Vision (n = 1) NCT03814824		Intelligent Real Time Image Segmentation (n = 1) NCT03814824
Other (n = 19)	Integrated AI System (n = 2) NCT03511690, NCT03999177		Intelligent System (n = 2) NCT03511690, NCT03999177
	Artificial Intelligence not specified (n = 17) NCT06182332, NCT05096286, NCT06305364, NCT05963724, NCT06888089, NCT05826288, NCT03925337, NCT03954548, NCT05113927, NCT03953976, NCT05030454, NCT05611151, NCT04411810, NCT05275556, NCT03620071, NCT04754347, NCT03867409		
Observational Trials			
Learning (n = 15)	Machine Learning (n = 15) NCT04441775, NCT05147389, NCT06463860, NCT06463977, NCT03688906, NCT04442425, NCT05385718, NCT03837327, NCT03174574, NCT04369053, NCT06381583, NCT05383976, NCT05122247, NCT06576232, NCT05126173		Convolutional Neural Network (n = 1) NCT05147389
			Deep Ensemble (n = 1) NCT05126173
			Deep Neural Network (n = 1) NCT06463860
Communication (n = 1)	Natural Language Processing (n = 1) NCT04442425		
Other (n = 8)	Artificial Intelligence not specified (n = 8) NCT04441775, NCT05147389, NCT03837327, NCT04369053, NCT06561217, NCT06576232, NCT05126173, NCT05872503		

* Some trials were classified with more than one choice possible based on the corresponding ClinicalTrials.gov entry.

**Table 4:** Summary.

CCC Domain	NCI Subcategory
Etiology	Environmental factors Genetic factors Gene–environment interactions Medication/pharmaceutical exposure Infectious agents Health behaviors
Prevention	Tobacco control Diet Physical activity Sun protection HPV vaccine Limited alcohol use Chemoprevention
Detection	Pap/HPV testing Mammography Fecal occult blood test Colonoscopy Lung cancer screening
Diagnosis	Shared and informed decision making
Treatment	Curative treatment Non-curative treatment Adherence Symptom management
Survivorship	Coping Health Promotion for Survivors

**Table 5:** Characteristics of AI-related interventional and observational oncology trials.

NCT # (Start Year, N)	Cancer Type	Cancer Control Continuum Term	AI Application and Implementation
Interventional			
NCT03511690 (2017, n = 95)	Multiple or Unspecified	Etiology (Genetic factors)	AI-driven avatar-based tutoring system.
NCT03063619 (2018, n = 194)	Breast	Prevention (Chemoprevention)	ML-assisted volumetric breast density measurement.
NCT03371147 (2018, n = 30)	Multiple or Unspecified	Treatment (Symptom Management)	NLP (sentiment analysis)-based digital platform (CancerLife) collects patient-reported symptoms and psychosocial data.
NCT03746392 (2018, n = 150)	Multiple or Unspecified	Survivorship (Coping)	ML and NLP analyze Electronic Health Records (EHRs) to prompt goals-of-care conversations.
NCT03867409 (2018, n = 2105)	Colorectal	Detection (Colonoscopy)	Culturally tailored messages delivered through AI-based virtual technology to increase Colorectal (CRC) screening.
NCT04277650 (2018, n = 311)	Multiple or Unspecified	Treatment (Symptom Management)	ML identifies high-risk individuals undergoing outpatient radiation or chemoradiation for targeted evaluations.
NCT03620071 (2019, n = 67)	Multiple or Unspecified	Survivorship (Coping)	GoalKeeper (Intelligent Information Sharing) [39] leverages AI to coordinate care via a mobile health platform.
NCT03814824 (2019, 148)	Esophagus	Detection (Endomicroscopy)	Intelligent Real-time AI image segmentation improves Volumetric Laser Endomicroscopy by flagging dysplastic regions.
NCT03925337 (2019, n = 234)	Colorectal	Detection (Colonoscopy)	Computer Aided Detection (CAD) DL software processes colonoscopy video in real-time.
NCT03953976 (2019, n = 67)	Head and Neck	Treatment (Curative Treatment)	AI-driven radiomic analysis of imaging scans guides precise targeting of involved lymph nodes.
NCT03984773 (2019, n = 78)	Multiple or Unspecified	Survivorship (Coping)	ML predicts patients at high risk of short-term mortality, prompting serious illness conversations.
NCT03999177 (2019, n = 30)	Breast	Survivorship (Health promotion for survivors)	Integrated AI-driven motion sensing guides lymphatic exercises.
NCT04195646 (2019, n = 300)	Colorectal	Detection (Colonoscopy)	EndoVigilant CAD processes colonoscopy video in real-time.
NCT03954548 (2020, n = 249)	Colorectal	Detection (Colonoscopy)	CNN based CAD device (GI-Genius) analyzes real-time colonoscopy videos.
NCT04411810 (2020, n = 149)	Skin	Diagnosis (Shared and Informed Decision Making)	AI-based Nevisense measures electrical impedance of skin lesions to assess malignancy.
NCT04551105 (2020, n = 16)	Breast	Diagnosis (Shared and Informed Decision Making)	DL analyzes multi-site breast ultrasound images.
NCT04458168 (2021, n = 120)	Gynecologic	Survivorship (Coping)	ML-based App, Purposeful, customizes app resources.
NCT04754347 (2021, n = 1472)	Colorectal	Detection (Colonoscopy)	CAD device (Skout) analyses colonoscopy video.
NCT04867850 (2021, n = 4450)	Multiple or Unspecified	Survivorship (Coping)	ML predicts 6-month mortality risk.
NCT05030454 (2021, n = 10)	Multiple or Unspecified	Treatment (Curative Treatment)	AI-powered ETHOS system integrates CT-guided adaptive radiotherapy and optical surface imaging.
NCT05069519 (2021, n = 126)	Multiple or Unspecified	Survivorship (Health promotion for survivors)	NLP sentiment analysis guides personalized exercise and social support from Fitbit and Facebook.
NCT05113264 (2021, n = 60)	Gastrointestinal	Treatment (Adherence)	NLP-based chatbot “Penny” triages patient-reported symptoms.
NCT05113927 (2021, n = 482)	Breast	Treatment (Curative treatment)	AI (SELENE) system provides real-time intraoperative imaging of lumpectomy margins.
NCT05096286 (2022, n = 10)	Multiple or Unspecified	Treatment (Non-curative treatment)	AI ETHOS system automates MRI image processing.
NCT05275556 (2022, n = 1410)	Colorectal	Detection (Colonoscopy)	AI processes White Light Endoscopy colonoscopy video.
NCT05963724 (2022, n = 1100)	Colorectal	Detection (Colonoscopy)	AI (GI-Genius) analyzes colonoscopy video.
NCT06621225 (2022, n = 264)	Colorectal	Detection (Colonoscopy)	ML (CNN) analyzes real-time colonoscopy images.
NCT04535414 (2023, n = 195)	Gastrointestinal	Detection (Endoscopy)	ML assists detection of early signet ring cell carcinoma.
NCT05611151 (2023, n = 830)	Colorectal	Detection (Colonoscopy)	ML-based CAD system, WISE VISION, processes white light colonoscopy images in real time.
NCT05826288 (2023, n = 11)	Blood	Survivorship (Health promotion for survivors)	AI monitoring supports post-BMT and CAR-T home care.
NCT06182332 (2023, n = 221)	Gynecologic	Survivorship (Coping)	AI analyzes EHR to identify advanced gynecologic cancer patients for outpatient palliative care.
NCT06305364 (2023, n = 426)	Colorectal	Detection (Colonoscopy)	Gixam device uses AI-analyzed tongue images to predict colorectal adenomas.
NCT06888089 (2023, n = 20,707)	Multiple or Unspecified	Treatment (Adherence)	ML combines genomic, clinical data, and NLP of imaging reports to identify trial-eligible patients.
Observational			
NCT03174574 (2016, n = 3)	Multiple or Unspecified	Etiology (Gene-environment interactions)	ML analyzes genetic mutation data.
NCT03688906 (2018, n = 3275)	Colorectal	Detection (Biomarkers)	ML analyzes multi-omics blood biomarkers to develop a non-invasive assay for early CRC detection.
NCT03837327 (2019, n = 1025)	Gynecologic	Diagnosis (Shared and Informed Decision Making)	ML analyzes glycoprotein patterns to distinguish benign from malignant adnexal masses.
NCT04441775 (2020, n = 5)	Prostate	Treatment (Curative)	ML generates standardized radiation treatment plans.
NCT05147389 (2020, n = 170)	Bile Duct	Diagnosis (Shared and Informed Decision Making)	ML (CNN) processes live cholangioscopy video footage.
NCT04442425 (2020, n = 83)	Multiple or Unspecified	Survivorship (Coping)	ML and NLP analyze facial, voice, and language cues to classify cancer-related pain.
NCT04369053 (2020, n = 48,995)	Colorectal	Detection (Biomarkers)	AI analyzes multi-omic patterns of cell-free biomarkers to develop a non-invasive blood assay for CRC detection.
NCT05385718 (2022, n = 694)	Multiple or Unspecified	Detection (MRI Imaging)	ML enhances MRI images for cancer detection.
NCT05383976 (2022, n = 201)	Colorectal	Detection (Colonoscopy; Fecal Immunochemical Test)	ML risk-stratifies patients in poverty-affected zip codes to increase CRC screening.
NCT05122247 (2022, n = 12,000)	Multiple or Unspecified	Treatment (Adherence; Symptom Management)	ML analyzes clinical data to predict unplanned hospital admissions and emergency visits.
NCT06576232 (2022, n = 1147)	Lung	Detection (Lung Cancer Screening)	ML analyzes low-dose CT images to support lung cancer screening.
NCT05126173 (2022, n = 1111)	Skin	Detection (Skin Cancer Screening)	Deep ensemble analyzes dermoscopic images.
NCT06463977 (2023, n = 52)	Lung	Survivorship (Coping)	ML generates individualized mortality risk predictions based on clinical data.
NCT06381583 (2023, n = 658)	Esophagus	Detection (Biomarkers)	ML identifies blood microRNA biomarkers for esophageal disease staging.
NCT06561217 (2023, n = 355)	Multiple or Unspecified	Treatment (Adherence)	Mendel uses AI to identify patients for clinical trials.
NCT06463860 (2024, n = 81)	Skin	Diagnosis (Shared and Informed Decision Making)	DermDx uses DL to analyze dermoscopic images.
NCT05872503 (2024, n = 3)	Prostate	Detection (MRI Imaging)	AI processes MRI scans to detect prostate cancer.

## Data Availability

No new data were created or analyzed in this study. The data supporting the findings of this work are derived from publicly available clinical trial records accessible at ClinicalTrials.gov (https://clinicaltrials.gov).

## Supplementary Materials

The following supporting information can be downloaded at: https://www.mdpi.com/article/10.3390/cancers17213537/s1. Table S1. Characteristics of AI-related interventional and observational oncology trials.

## Abbreviations

The following abbreviations are used in this manuscript:

AI	Artificial Intelligence
CCC	Cancer Control Continuum
ML	Machine Learning
NLP	Natural Language Processing
DL	Deep Learning
N/A	Not Applicable
FIT	Fecal Immunochemical Testing
CNN	Convolutional Neural Network
EHR	Electronic Health Record
CRC	Colorectal Cancer
CAD	Computer-Aided Detection
BRCA Gist	BReast CAncer Genetics Intelligent Semantic Tutoring
PCP	Primary Care Physician
