# Supplementary material for: Artificial Intelligence in Oncology: A 10-Year ClinicalTrials.gov-Based Analysis Across the Cancer Control Continuum
Source: Cancers (Basel). 2025 Nov 1;17(21):3537. doi: 10.3390/cancers17213537 (PMC12607805; doi:10.3390/cancers17213537)
Supplement: Supplementary file 1 [file cancers-17-03537-s001.zip › cancers-3961661-supplementary.pdf]

Supplementary Materials

Supplementary Materials:

The following search terms were applied.

Artificial Intelligence" OR "AI System*" OR "Intelligent Systems" OR "Machine Learning" OR "deep learning" OR autoencoder OR "large language model*" OR "dimensionality reduction" OR "ensemble learning", "deep ensemble" OR "federated learning" OR "multiple instance learning algorithm*" OR "support vector machine*" OR "particle swarm optimization" OR "Machine Pattern Analyses" OR "Machine Pattern Analysis" OR "trend analysis" OR "trend analyses" OR "machine pattern recognition" OR "machine pattern detection*" OR "Machine Prediction Method*" OR "Predictive Learning Model*" OR "Machine Predictive Model*" OR "Sentiment Analysis" OR "sentiment analyses" OR "sentiment classification*" OR "opinion mining" OR "natural language processing" OR "Computer Neural Network*" OR "Neural Network Model*" OR "Connectionist Model*" OR "Computational Neural Network*" OR "Convolutional Neural Network*" OR LeNet OR AlexNet OR VGGNet OR VGGNets OR "Visual Geometry Group Network" OR ZFNet OR ZFNets OR GoogLeNet OR GoogLeNets OR MobileNets OR "Residual Neural Network*" OR ResNets OR EfficientNet OR "Densely Connected Convolutional Network*" OR DenseNet OR "neural network*" OR "Extreme Learning Machine*" OR "Multilayer Perceptron*" OR "Multi-Layer Perceptron*" OR "Radial Basis Function Network*" OR "Radial Basis Network*" OR "Radial Basis Function*" OR "Long Short Term Memory" OR "Ising Model" OR Chat-GPT OR "Chat GPT" OR ChatGPT OR ChatGPTs OR "ambient intelligence" OR "artificial general intelligence" OR "artificial superintelligence" OR "automated reasoning" OR "machine reasoning" OR "gen-ai" OR "genai" OR "generative a.i." OR "generative ai" OR "generative pre-trained transformer" OR "generative pre-training transformer" OR "generative pretrained transformer"

Supplementary Table S1: Characteristics of AI-Related Interventional and Observational Oncology Trials

NCT #	Start Year	Enrollment Size	Cancer Type	Cancer Control Continuum Term	AI Application	FDA Regulation Status	Implementation	Key Findings from posted results or associated publications
**Interventional Trials**								
NCT03511690<br><br>https://clinicaltrials.gov/study/NCT03511690	2017	95	Multiple or Unspecified	Etiology (Genetic factors)	Integrated Artificial Intelligence System	N/A	A web and artificial intelligence-driven avatar-based tutoring system delivers tailored, culturally sensitive education on hereditary breast and ovarian cancer risk, enhancing knowledge and psychosocial outcomes for underserved Black and Latina women.	**Associated publications** do not provide trial results.<br><br>This may reflect foundational research or studies that are not directly related to this specific trial.
NCT03063619<br><br>https://clinicaltrials.gov/study/NCT03063619	2018	194	Breast	Prevention (Chemoprevention)	Machine Learning	N/A	Artificial intelligence assisted volumetric breast density measurement using the Volpara software enables precise quantification of mammographic density changes over time, supporting evaluation of afimoxifene's efficacy in reducing breast cancer risk in women with dense breasts.	**Posted Results:** Volpara (ML based tool) produced more consistent and less variable measurements, while Cumulus (semi-automated tool) was more sensitive to larger changes in breast density.
NCT03371147<br><br>https://clinicaltrials.gov/study/NCT03371147	2018	30	Multiple or Unspecified	Treatment (Symptom Management)	Natural Language Processing (Sentiment analysis)	N/A	A standalone digital platform (CancerLife) collects patient-reported symptoms and psychosocial data shared with caregivers and care teams via existing social networks.	N/A

NCT03746392<br><br>https://clinicaltrials.gov/study/NCT03746392	2018	150	Multiple or Unspecified	Survivorship (Coping)	Machine Learning; Natural Language Processing	N/A	Machine learning and natural language processing analyze electronic health records to identify prior goals-of-care discussions and advance directives, generating personalized forms ("Jumpstart") that guide and prompt clinicians and patients to engage in timely goals-of-care conversations.	**Associated Publication:** The communication-priming intervention increased EHR-documented goals-of-care discussions from 8% to 21% in hospitalized seriously ill patients, showing feasibility and acceptability, though patient-reported discussions and communication quality remained unchanged due to complex inpatient dynamics.
NCT03867409<br>https://clinicaltrials.gov/study/NCT03867409	2018	2105	Colorectal	Detection (Colonoscopy)	Artificial Intelligence (Virtual Human Technology)	N/A	Culturally sensitive, tailored interactive messages delivered through virtual humans or text-based platforms to enhance knowledge, attitudes, and behaviors toward colorectal cancer screening among diverse populations.	N/A
NCT04277650<br><br>https://clinicaltrials.gov/study/NCT04277650	2018	311	Multiple or Unspecified	Treatment (Symptom Management)	Machine Learning	N/A	Machine learning algorithm analyzes patient data to identify high-risk individuals undergoing outpatient radiation or chemoradiation, enabling targeted clinical evaluations twice-weekly for high-risk patients and once-weekly for others to reduce emergency visits and hospitalizations effectively.	**Associated Publication:** Machine learning accurately identified high-risk radiation therapy patients; twice-weekly artificial intelligence-guided evaluations reduced acute care visits from 22.3% to 12.3%, lowered preventable emergencies, and decreased hospitalizations, demonstrating effective clinical triage and potential healthcare cost savings.

NCT03620071<br><br>https://clinicaltrials.gov/study/NCT03620071	2019	67	Multiple or Unspecified	Survivorship (Coping)	Artificial Intelligence (Intelligent Information Sharing)	N/A	GoalKeeper employs artificial intelligence within a mobile health platform to facilitate communication and coordination among multiple caregivers. It supports goal-centered care planning by aggregating input from parents, healthcare providers, and community resources to enhance care for children with medical complexity.	**Associated Publication**: Family-centered goal-setting tool (AI-based GoalKeeper)[1] was feasible and acceptable among providers and parents of children with medical complexity; email reminders and shared goal setting facilitated use, but lack of EHR integration limited sustained engagement.
NCT03814824<br><br>https://clinicaltrials.gov/study/NCT03814824	2019	148	Esophagus	Detection (Endomicroscopy)	Computer Vision (Intelligent Real-Time Image Segmentation)	FDA Regulated Device	The intelligent real-time image segmentation AI-assisted system enhances volumetric laser endomicroscopy (VLE) by automatically highlighting areas suspicious for dysplasia during exams, improving targeted biopsies and reducing interpretation time for clinicians.	**Associated Publication and Posted Results**: Volumetric laser endomicroscopy (VLE) with Intelligent Real-time Image Segmentation increased interpretation time compared to VLE alone; achieved the highest dysplasia (precancerous cell changes) detection rate, slightly exceeding VLE alone and the Seattle protocol (standardized method of biopsy collection).
NCT03925337<br><br>https://clinicaltrials.gov/study/NCT03925337	2019	234	Colorectal	Detection (Colonoscopy)	Artificial Intelligence (Deep Learning)	N/A	Artificial intelligence software processes colonoscopy video in near real-time on a separate monitor, highlighting potential polyps to aid detection beyond standard colonoscopy.	**Associated Publication**: A deep learning artificial intelligence system analyzes colonoscopy video in near real-time to detect colonic polyps and adenomas by highlighting suspicious

								lesions, aiming to reduce misses, improve detection rates, and lower colorectal cancer risk through enhanced accuracy.
NCT03953976<br><br>https://clinicaltrials.gov/study/NCT03953976	2019	67	Head and Neck	Treatment (Curative Treatment)	Artificial Intelligence	N/A	Artificial intelligence-driven radiomic analysis of imaging scans (CT, PET-CT, MRI) guides precise targeting of involved lymph nodes during radiation therapy for head and neck squamous cell carcinoma, aiming to minimize radiation exposure, reduce toxicity, and optimize treatment effectiveness while preserving healthy tissue.	N/A
NCT03984773<br><br>https://clinicaltrials.gov/study/NCT03984773	2019	78	Multiple or Unspecified	Survivorship (Coping)	Machine Learning	N/A	The machine learning model was used to accurately predict patients at high risk of short-term mortality, enabling timely identification of individuals who could benefit from serious illness conversations. This facilitated targeted behavioral nudges to clinicians, promoting advance care planning discussions aligned with patient needs.	**Associated Publication:** Nudging intervention using machine learning prompts increased serious illness conversation documentation overall and among high-risk patients; the intervention also improved patient-clinician relationship discussions but reduced discussion of family involvement.
NCT03999177<br><br>https://clinicaltrials.gov/study/NCT03999177	2019	30	Breast	Survivorship (Health promotion for survivors)	Integrated Artificial Intelligence System	N/A	The Kinect- The Optimal Lymphatic Flow (TOLF) system employs motion-sensing technology to monitor and guide patients in	**Associated Publication:** The Kinect-TOLF training system demonstrated 100% feasibility, high usability (>90% user satisfaction),

							performing lymphatic exercises accurately, providing real-time feedback that enhances patient confidence and self-efficacy in managing lymphedema symptoms effectively.	and significant immediate reductions in lymphatic pain, swelling, lymphedema symptoms, symptom severity, and lymph fluid levels among breast cancer survivors
NCT04195646<br><br>https://clinicaltrials.gov/study/NCT04195646	2019	300	Colorectal	Detection (Colonoscopy)	Machine Learning (Deep Learning)	FDA Regulated and Unapproved/Uncleared Device	EndoVigilant Computer Aided Detection (CAD) software processes colonoscopy video in real-time and provides an annotated video on a second monitor highlighting polyps and adenomas, enabling better detection during the procedure.	N/A
NCT03954548<br><br>https://clinicaltrials.gov/study/NCT03954548	2020	249	Colorectal	Detection (Colonoscopy)	Artificial Intelligence<br><br>*Intervention described in the associated publication as using a convolutional neural network, though this was not specified in the ClinicalTrials.gov entry.	FDA Regulated Device	Utilized a convolutional neural network based real-time computer-aided detection (GI-Genius) system. Artificial intelligence analyzes real-time colonoscopy videos, highlighting suspicious lesions to reduce missed adenomas, with output integrated directly on primary monitor.	**Associated Publication:** Artificial intelligence-assisted colonoscopy reduced adenoma miss rate from 32.4% to 15.5%, decreased false negatives from 29.6% to 6.8%, and improved detection of small, flat, proximal, and distal lesions.
NCT04411810	2020	149	Skin	Diagnosis (Shared and Informed	Artificial Intelligence	FDA Regulated Device	Nevisense measures electrical impedance of skin lesions to assess	**Posted Results:** Nevisense (AI diagnostic tool) assisted telemedicine demonstrated

https://clinicaltrials.gov/study/NCT04411810				Decision Making)			malignancy risk, while Dermlite Cam and Barco Demetra capture high-quality clinical and dermoscopic images, collectively enabling remote dermatologists to make informed diagnostic and biopsy decisions via telemedicine.	lower accuracy in skin cancer diagnosis compared to in-person dermatologist assessment and telemedicine without Nevisense.
NCT04551105<br><br>https://clinicaltrials.gov/study/NCT04551105?cond=NCT04551105	2020	16	Breast	Diagnosis (Shared and Informed Decision Making)	Machine Learning (Deep Learning)	FDA Regulated and Unapproved/Uncleared Device	Deep learning was used to analyze breast ultrasound images across multiple cases, assisting radiologists by highlighting lesions and providing decision support that enhanced diagnostic accuracy, inter-reader agreement, and efficiency during clinical evaluations.	**Posted Results:** The deep learning system improved breast ultrasound diagnosis by increasing accuracy, enhancing sensitivity and specificity, and reducing reading time, thereby aiding faster, more accurate lesion identification and supporting better clinical decisions.
NCT04458168<br><br>https://clinicaltrials.gov/study/NCT04458168	2021	120	Gynecologic	Survivorship (Coping)	Machine Learning		The machine learning based eHealth App, Purposeful, customizes app resources and facilitates patient reflections to enhance purposeful living through daily interactive self-care activities and habit tracking.	N/A
NCT04754347<br><br>https://clinicaltrials.gov/study/NCT04754347	2021	1472	Colorectal	Detection (Colonoscopy)	Artificial Intelligence<br><br>*Intervention described in the associated publication as using a deep neural network,	FDA Regulated and Unapproved/Uncleared Device	The Skout computer aided detection device performs automated real-time analysis of colonoscopy video, highlighting potential colorectal polyps to assist endoscopists during procedures.	**Associated Publication:** Use of the computer aided detection device significantly increased adenomas detected per colonoscopy compared to standard colonoscopy, while maintaining a statistically

					though this was not specified in the ClinicalTrials.gov entry.			non-inferior true histology rate.
NCT04867850<br><br>https://clinicaltrials.gov/study/NCT04867850	2021	4450	Multiple or Unspecified	Survivorship (Coping)	Machine Learning		Machine learning model predicts 6-month mortality risk; behavioral nudges (performance feedback, text reminders, patient priming) are also used to optimize serious illness conversation documentation.	**Associated Publication and Posted Results:** Artificial intelligence driven behavioral nudges substantially increased serious illness conversation documentation among high-risk cancer patients. The combined approach targeting both clinicians and patients was most effective, enhancing communication, aligning care with patient preferences, and reducing disparities across racial and ethnic groups for more equitable care.
NCT05030454<br><br>https://clinicaltrials.gov/study/NCT05030454	2021	10	Multiple or Unspecified	Treatment (Curative Treatment)	Artificial Intelligence	FDA Regulated and Unapproved/Uncleared Device	The artificial intelligence powered ETHOS system combines CT-guided stereotactic adaptive radiotherapy with real-time optical surface imaging, using on-board cone beam CT for adaptive planning and intra-fraction motion control, enabling precise and efficient treatment delivery in respiratory-motion-affected mobile regions during	**Posted Results:** Artificial intelligence driven surface guidance integrated with CT-based adaptive radiotherapy enabled precise motion management during treatment of mobile upper abdomen and thorax tumors, achieving 100% successful radiation delivery fractions, reducing setup variability, minimizing treatment

							Stereotactic Body Radiotherapy (SBRT).	interruptions, and improving targeting accuracy.
NCT05069519<br><br>https://clinicaltrials.gov/study/NCT05069519	2021	126	Multiple or Unspecified	Survivorship (Health promotion for survivors)	Natural Language Processing (Sentiment analysis)		Personalized exercise prescriptions based on Fitbit data and Facebook health education; sentiment and behavior analysis guides feedback and social support.	N/A
NCT05113264<br><br>https://clinicaltrials.gov/study/NCT05113264	2021	60	Gastrointestinal	Treatment (Adherence)	Natural Language Processing		Algorithmically driven augmented intelligence chatbot “Penny” uses predefined clinical guidelines to triage patient-reported symptoms, guide self-care for low-grade side effects, and escalate severe symptoms to healthcare teams via automated alerts, supporting medication adherence and toxicity management	N/A
NCT05113927<br><br>https://clinicaltrials.gov/study/NCT05113927	2021	482	Breast	Treatment (Curative treatment)	Artificial Intelligence	FDA Regulated and Unapproved/Uncleared Device	Artificial intelligence assisted wide field Optical Coherence Tomography (SELENE system) provides real-time intraoperative imaging of lumpectomy margins, enabling surgeons to assess and potentially resect positive margins during breast conservation surgery, aiming to reduce residual tumor presence and improve surgical outcomes.	N/A

NCT05096286<br><br>https://clinicaltrials.gov/study/NCT05096286?term=NCT05096286	2022	10	Multiple or Unspecified	Treatment (Non-curative treatment)	Artificial Intelligence	FDA Regulated Device	Artificial intelligence assisted semi-automated processing of diagnostic MRI data within the Ethos Therapy system enables on-table adaptive planning for positioning adjustments, facilitating faster simulation-free Hippocampal Avoidance Whole-Brain Radiotherapy delivery with comparable plan quality, preserved cognitive function, improved workflow efficiency, and demonstrated safety and feasibility.	N/A
NCT05275556<br><br>https://clinicaltrials.gov/study/NCT05275556	2022	1410	Colorectal	Detection (Colonoscopy)	Artificial Intelligence	FDA Regulated and Unapproved/Uncleared Device	AI processes HD White Light Endoscopy colonoscopy video to detect and highlight potential colorectal polyps using graphical overlays without altering video feed.	**Posted Results:** Computer-Assisted Detection (CADe) Device colonoscopy significantly increased adenoma detection rate compared to standard High-Definition White Light Endoscopy Colonoscopy; did not demonstrate non-inferiority for detection of clinically significant lesions.
NCT05963724<br><br>https://clinicaltrials.gov/study/NCT05963724?term=NCT05963724	2022	1100	Colorectal	Detection (Colonoscopy)	Artificial Intelligence	FDA Regulated Device	Artificial Intelligence based real-time analysis of colonoscopy video framed by the GI-Genius system to detect colonic polyps, enhancing endoscopist detection accuracy during procedures.	**Associated Publication:** Microsimulation modeling shows real-time computer aided detection using GI-Genius is cost-effective if it increases adenoma detection from 26% to ≥30% or costs less than $579 per

								use, highlighting its potential economic and clinical impact in routine colonoscopy practice.
NCT06621225<br><br>https://clinicaltrials.gov/study/NCT06621225	2022	264	Colorectal	Detection (Colonoscopy)	Machine Learning (Convolutional neural network)	FDA Regulated Device	AI employs a convolutional neural network-based model to analyze real-time colonoscopy images, detecting and highlighting polyps, while a mucosal exposure device (EndoCuff Vision) enhances visualization for more accurate lesion identification during procedures.	**Associated publications** do not provide trial results.<br><br>This may reflect foundational research or studies that are not directly related to this specific trial.
NCT04535414<br><br>https://clinicaltrials.gov/study/NCT04535414	2023	195	Gastrointestinal	Detection (Endoscopy)	Machine Learning	FDA Regulated Device	Participants underwent confocal endomicroscopy of gastric mucosa with machine learning algorithms assisting in detection of early signet ring cell carcinoma comparing the Bethesda Protocol to the Cambridge Method.	**Posted Results:** In this early-phase study of CDH1 mutation carriers, the Bethesda Protocol showed a higher crude cancer detection rate on endoscopy compared to the Cambridge Method. Among the two Bethesda participants who underwent gastrectomy, one false negative occurred, while no participants in the Cambridge arm underwent surgery.
NCT05611151<br><br>https://clinicaltrials.gov/study/NCT05611151	2023	830	Colorectal	Detection (Colonoscopy)	Artificial Intelligence; Machine Learning<br><br>*Intervention described in the associated	FDA Regulated and Unapproved/Uncleared Device	The artificial intelligence/machine learning-based computer aided detection system, WISE VISION, processes high-definition white light colonoscopy images in real	**Associated Publication:** The deep learning AI system achieved 97.3% sensitivity and 99.0% specificity in detecting colorectal lesions, including challenging non-polypoid polyps, with real-

					publication as using deep learning, though this was not specified in the ClinicalTrials.gov entry.		time, assisting endoscopists by detecting and highlighting colorectal lesions during procedures to improve accuracy and facilitate immediate clinical decision-making.	time processing, enhancing endoscopist detection accuracy and potentially reducing missed colorectal cancers during colonoscopy.
NCT05826288<br><br>https://clinicaltrials.gov/study/NCT05826288	2023	11	Blood	Survivorship (Health promotion for survivors)	Artificial Intelligence	N/A	Artificial intelligence-assisted remote physiological monitoring combined with automated messaging supports home care and timely interventions for post-Bone Marrow Transplant and Chimeric Antigen Receptor T-cell therapy patients, enhancing care coordination and early complication detection; this study evaluates its feasibility and patient-provider experience.	N/A
NCT06182332<br><br>https://clinicaltrials.gov/study/NCT06182332?term=NCT06182332	2023	221	Gynecologic	Survivorship (Coping)	Artificial Intelligence	N/A	Weekly analysis of electronic health records using an artificial intelligence risk-scoring algorithm identifies advanced gynecologic cancer patients who may benefit from outpatient palliative care, enabling timely referrals that improve symptom management and ensure care aligns with patients' preferences and goals.	N/A

NCT06305364<br><br>https://clinicaltrials.gov/study/NCT06305364?term=NCT06305364	2023	426	Colorectal	Detection (Colonoscopy)	Artificial Intelligence	FDA Regulated and Unapproved/Uncleared Device	The Gixam device captures tongue images and are analyzed by an artificial intelligence model to predict colorectal adenomas with promising accuracy, enabling potential non-invasive early detection; diagnostic accuracy is validated against standard colonoscopy and pathology findings.	N/A
NCT06888089<br><br>https://clinicaltrials.gov/study/NCT06888089?term=NCT06888089	2023	20707	Multiple or Unspecified	Treatment (Adherence)	Artificial Intelligence; Machine Learning; Natural Language Processing	N/A	Machine learning models integrate structured genomic and clinical data with natural language processing of imaging reports to improve oncologist awareness of trial-eligible patients, potentially enhancing clinical trial enrollment and accelerating cancer treatment development.	N/A
**Observational**								
NCT03174574 https://clinicaltrials.gov/study/NCT03174574	2016	3	Multiple or Unspecified	Etiology (Gene-environment interactions)	Machine Learning	N/A	Machine learning models analyze genetic mutations (CDKN2A) alongside clinical and environmental data from families with pancreatic cancer and melanoma to identify modifiers of cancer susceptibility, tumor behaviors, and gene-	N/A

							environment interactions influencing disease expression.	
NCT03688906 https://clinicaltrials.gov/study/NCT03688906	2018	3275	Colorectal	Detection (Biomarkers)	Machine Learning	N/A	Machine learning analyzes multi-omics patterns of cell-free biomarkers in blood samples to develop a non-invasive, blood-based assay aimed at early detection of colorectal cancer and advanced adenomas, enhancing screening accuracy and patient convenience.	**Associated Publication**: Multiomics ML-Based colorectal cancer test demonstrated high early-stage sensitivity in adenocarcinoma; broader subtype detection remains limited.
NCT03837327<br><br>https://clinicaltrials.gov/study/NCT03837327	2019	1025	Gynecologic	Diagnosis (Shared and Informed Decision Making)	Artificial Intelligence; Machine Learning	N/A	Artificial intelligence and mass spectrometry analyze blood-based glycoprotein patterns to distinguish benign from malignant adnexal masses, aiming to validate ovarian cancer-specific signatures.	N/A
NCT04441775<br><br>https://clinicaltrials.gov/study/NCT04441775	2020	5	Prostate	Treatment (Curative)	Artificial Intelligence; Machine Learning	N/A	Artificial intelligence models are trained on expert-curated prostate cancer radiation treatment plans from multiple institutions, processing patient CT scans to generate standardized radiation treatment plans that maintain therapeutic targets while respecting dose constraints, aiming to improve consistency and quality in oncology radiation planning.	N/A

NCT05147389<br><br>https://clinicaltrials.gov/study/NCT05147389	2020	170	Bile Duct	Diagnosis (Shared and Informed Decision Making)	Artificial Intelligence; Machine Learning (Convolutional Neural Network)	N/A	The artificial intelligence model processes live digital single-operator cholangioscopy video footage, detecting and highlighting regions with malignancy features in real time. It aids endoscopists by improving the identification and classification of neoplastic versus non-neoplastic bile duct lesions during procedures.	**Associated publications** report results from different clinical trials (NCT02794987, NCT02166099), not the listed NCT05147389.<br><br>This may reflect foundational research or studies that are not directly related to this specific trial.
NCT04442425 https://clinicaltrials.gov/study/NCT04442425	2020	83	Multiple or Unspecified	Survivorship (Coping)	Machine Learning; Natural Language Processing	N/A	The artificial intelligence system integrates machine learning and natural language processing to analyze facial expressions, voice signals, and spoken language from videos and audio recordings, creating a multidimensional model that objectively detects and classifies cancer-related pain across diverse patient populations.	N/A
NCT04369053 https://clinicaltrials.gov/study/NCT04369053	2020	48995	Colorectal	Detection (Biomarkers)	Artificial Intelligence; Machine Learning	N/A	AI technology analyzes multi-omic patterns of cell-free biomarkers in blood samples, developing a non-invasive assay for early colorectal cancer detection, aiming to enhance screening accuracy, patient compliance, and facilitate timely diagnosis through	N/A

							advanced machine learning-based molecular profiling.	
NCT05385718<br><br>https://clinicaltrials.gov/study/NCT05385718	2022	694	Multiple or Unspecified	Detection (MRI Imaging)	Machine Learning	N/A	Machine learning enhances accelerated MRI sequences by restoring image quality to match traditional longer scans, enabling faster, cost-effective early cancer detection through improved imaging clarity in comprehensive, non-invasive MRI screening protocols.	N/A
NCT05383976<br><br>https://clinicaltrials.gov/study/NCT05383976	2022	201	Colorectal	Detection (Colonoscopy; (Fecal Immunochemical Test)	Machine Learning	N/A	Machine learning algorithm integrates with Penn Medicine's colorectal cancer patient navigation program to risk-stratify patients in poverty-affected zip codes, enabling prioritized, culturally sensitive outreach and navigation to increase timely colorectal cancer screening completion among high-risk populations.	N/A
NCT05122247<br><br>https://clinicaltrials.gov/study/NCT05122247	2022	12000	Multiple or Unspecified	Treatment (Adherence; Symptom Management)	Machine Learning	N/A	The validated machine learning model analyzes clinical data from chemotherapy patients to predict risk of unplanned hospital admissions and emergency visits, enabling proactive, personalized interventions that improve supportive care and reduce	**Associated publication** reports results from a different clinical trial (NCT04277650), not the listed NCT05122247.<br><br>This may reflect foundational research or studies that are not directly related to this specific trial.

							acute care utilization during outpatient cancer therapy.	
NCT06576232<br><br>https://clinicaltrials.gov/study/NCT06576232	2022	1147	Lung	Detection (Lung Cancer Screening)	Artificial Intelligence; Machine Learning	FDA Regulated and Unapproved/Uncleared Device	The artificial intelligence system employs machine learning to analyze chest low-dose CT images, performing end-to-end detection, localization, and characterization of pulmonary nodules, generating diagnostic reports to support clinicians in lung cancer screening and diagnosis workflows.	N/A
NCT05126173<br><br>https://clinicaltrials.gov/study/NCT05126173	2022	1111	Skin	Detection (Skin Cancer Screening)	Artificial Intelligence; Machine Learning (Deep Ensemble)	FDA Regulated and Unapproved/Uncleared Device	The deep ensemble algorithm is used to analyze dermoscopic images of suspicious skin lesions, accurately identifying melanoma, basal cell carcinoma, and squamous cell carcinoma, providing diagnostic support to clinicians for enhanced skin cancer detection and classification.	N/A
NCT06463977<br>https://clinicaltrials.gov/study/NCT06463977	2023	52	Lung	Survivorship (Coping)	Machine Learning	N/A	The machine learning model generates individualized mortality risk predictions based on clinical patient data; integrated into vignettes to evaluate effects on medical oncologists' prognostic accuracy and decision-making in advanced	N/A

							non-small cell lung cancer scenarios.	
NCT06381583 https://clinicaltrials.gov/study/NCT06381583	2023	658	Esophagus	Detection (Biomarkers)	Machine Learning	N/A	Machine learning algorithms analyze cell-free microRNA profiles from blood samples, identifying biomarker panels that differentiate early Barrett's esophagus from esophageal adenocarcinoma, enabling non-invasive, sensitive, and specific liquid biopsy screening for early cancer detection.	N/A
NCT06561217 https://clinicaltrials.gov/study/NCT06561217	2023	355	Multiple or Unspecified	Treatment (Adherence)	Artificial Intelligence	N/A	Mendel employs artificial intelligence to automatically extract and rank relevant clinical data from electronic health records, supporting human reviewers in accurately and efficiently identifying eligible oncology patients for clinical trial enrollment, optimizing patient selection workflows.	N/A
NCT06463860 https://clinicaltrials.gov/study/NCT06463860	2024	81	Skin	Diagnosis (Shared and Informed Decision Making)	Machine Learning (Deep Neural Network)	FDA Regulated and Unapproved/Uncleared Device	The DermDx deep learning algorithm analyzes dermoscopic images from commercial devices, providing diagnostic support to primary care physicians by enhancing detection and classification of skin cancers, aiming to improve diagnostic accuracy and sensitivity in clinical settings.	**Associated publications** do not provide trial results.

NCT05872503<br><br>https://clinicaltrials.gov/study/NCT05872503	2024	3	Prostate	Detection (MRI Imaging)	Artificial Intelligence	N/A	Artificial Intelligence algorithms process multiparametric MRI scans alongside clinical and genomic data to detect clinically significant prostate cancer in men of African ancestry, enhancing biopsy targeting and diagnostic precision to improve early detection and address racial disparities in prostate cancer outcomes.	N/A
